# A Crisis within a Crisis: COVID-19 and Hunger in African Children

**DOI:** 10.4269/ajtmh.20-1213

**Published:** 2020-11-06

**Authors:** Abdullahi Tunde Aborode, Samuel Olarewaju Ogunsola, Abidemi Olugbenga Adeyemo

**Affiliations:** 1 Healthy Africans Platform, Research and Development, Ibadan, Nigeria;; 2 Food and Genes Initiative, Research and Development, Yaba, Lagos, Nigeria;; 3 Centre for Integrated Health Programs, ART Clinic, Lagos University Teaching Hospital, Lagos, Nigeria;; 4 Department of Public Health, University of Nicosia Medical School, Nicosia, Cyprus

## Abstract

The WHO recently expressed concern at the potential impact of COVID-19 on hunger, which is likely to exacerbate the already considerable burden of malnutrition of Africa. The impact of the disease is expected to be greater among those grappling with malnutrition, whereas widespread hunger and malnutrition will likely increase because of movement restrictions. COVID-19 is unfolding in Africa against a backdrop of worrying levels of hunger and undernourishment which could worsen as the virus threatens livelihoods and household economies. The perspective piece addresses the crisis within crisis of COVID-19 and hunger on the well-being of children in Africa.

The advent of the COVID-19 pandemic has led to increase in nutritional challenges across the world, particularly in low-income and middle-income countries (LMICs).^1^ The demography most affected are the young children between ages 1–5 years in these regions. As a means of curbing the pandemic, strategies such as physical distancing, school closures, trade restrictions, and country lockdowns have been introduced. However, they have significantly increased food insecurity by affecting the production and sales of nutritional and affordable food products, which have led to millions of families relying on nutrient-poor alternatives. Strained health systems and interruptions in humanitarian response are eroding access to essential and often life-saving nutrition services.^2^ Social protection systems in many LMICs are overloaded as vulnerable families struggle to access the food and services they need in the context of an economic downturn.

In the course of the COVID-19 pandemic, a recent modeling exercise using estimates of potential impacts of COVID-19–related economic deterioration, food insecurity, and interruption of community-based malnutrition programs suggests that the prevalence of wasting could increase by 10–50%, with an excess of 40,000–2,000,000 child deaths.^2^ Concurrently, more children are becoming malnourished because of the deterioration in the quality of their diets, interruptions in nutrition and other essential services, and the socioeconomic shocks created by the pandemic in LMICs.^3^ It was suggested that without timely action, the global prevalence of child wasting could rise by a shocking 14.3%.^3^

With an estimated of 47 million children younger than 5 years affected by wasting globally before the COVID-19 pandemic,^4^ this would translate to an estimated additional 6.7 million children with wasting during the first 12 months of the pandemic, 80% of them in sub-Saharan Africa and South Asia and more than 10,000 additional child deaths per month during this same period.^4^

With services for the prevention and treatment of wasting to a large extent up-ended in LMICs,^4^ millions of children are at risk of not receiving the care they need to survive and thrive. UNICEF reports from the early months of the COVID-19 pandemic suggest a 30% reduction in the coverage of essential nutrition services in LMICs and declines of 75–100% under lockdown contexts.^5^

UNICEF estimates that a minimum of US$2.4 billion is needed immediately to protect these children, prevent and treat malnutrition, and avoid human loss. There is an estimate of U$2.4 billion including an essential package of four life-saving interventions: prevention of wasting in children at risk, treatment for children who are wasted; biannual vitamin. A supplementation for children aged 6–59 months (90% coverage)^6^; and mass communication for the protection, promotion, and support of breastfeeding that focuses on caregivers or families of children aged 0–23 months.^7^

COVID-19 is making access to and availability of food more challenging for many worldwide, an undesirable situation that is likely to remain for the foreseeable future as an aftershock of the pandemic.^8^

The economic, food, and health system disruption resulting from the COVID-19 pandemic is expected to continue to exacerbate all forms of malnutrition.^9^ Estimates from the international food policy research institute suggest that because of the pandemic, an additional 140 million people will be thrown into living in extreme poverty on less than 1.90$ per day in 2020.^9^ According to the World Food Programme, the number of people in LMICs facing acute food insecurity will nearly double to 265 million by the end of 2020.^10^

Aside the estimated increase in marasmus, the COVID-19 pandemic will lead to increase in other forms of child malnutrition such as stunting, micronutrient deficiencies, and overweight. The lack of action from the global community will have a devastating long-term effect on young children, human productivity, and economy of many nations.

Protecting children’s right to nutrition in the face of the COVID-19 pandemic is highly needed at this time. This requires a swift response and investments from governments, donors, the private sector, and the United Nations. Five actions must be taken and tracked immediately.

Access to nutritious, safe, and affordable diets needs to be safeguarded and promoted as a cornerstone of the response to COVID-19. This can be performed by protecting food producers, processors, and retailers; discouraging trade bans; and designating food markets to be essential services and keeping them functioning and safe for workers and consumers.

Investments are needed to improve maternal and child nutrition through pregnancy, infancy, and early childhood by protecting breastfeeding and preventing the inappropriate marketing of infant formula in the context of COVID-19; securing children and women’s access to nutritious and diverse foods; and providing accurate information on infant feeding to caregivers.

Services for the early detection and treatment of child wasting need to be reactivated and scaled up while maintaining and expanding prevention and other nutrition services, including vitamin A supplementation for children, and micronutrient supplementation and nutritional support for pregnant and breastfeeding women, and minimizing the risk of infection.

Maintain the provision of nutritious and safe school meals for vulnerable children through home delivery, take-home rations, and cash or vouchers when schools are closed. These efforts also need to ensure adequate nutritional value of school meals or food packages and avoid the provision of unhealthy foods and beverages.

Finally, organizations working on providing social protections to safeguard access to nutritious diets and essential services among the poorest households should be supported. They should be assisted in creating interventions that will reach families with young children, and pregnant and breastfeeding women.

Globally, different nongovernmental organizations provide food palliatives as awareness on good feeding habits during the peak of the COVID-19 pandemic. Today, more than ever, all stakeholders including the government, private organizations, and civic societies must collaborate and act together to invest and create interventions that will help reduce the burden of malnutrition caused by the COVID-19 crisis in low- and middle-income countries.
